# A Skill‐Based Approach to Modeling the Attentional Blink

**DOI:** 10.1111/tops.12514

**Published:** 2020-07-17

**Authors:** Corné Hoekstra, Sander Martens, Niels A. Taatgen

**Affiliations:** ^1^ Bernoulli Institute for Mathematics, Computer Science, and Artificial Intelligence University of Groningen; ^2^ Department of Biomedical Sciences of Cells and Systems University Medical Center Groningen/Cognitive Neuroscience Center University of Groningen

**Keywords:** Attentional blink, PRIMs, ACT‐R, Skill‐based modeling, Cognitive model, Instruction learning, Skill‐based approach, Cognitive architectures

## Abstract

People can often learn new tasks quickly. This is hard to explain with cognitive models because they either need extensive task‐specific knowledge or a long training session. In this article, we try to solve this by proposing that task knowledge can be decomposed into skills. A skill is a task‐independent set of knowledge that can be reused for different tasks. As a demonstration, we created an attentional blink model from the general skills that we extracted from models of visual attention and working memory. The results suggest that this is a feasible modeling method, which could lead to more generalizable models.

## Abstract 2

People can learn to perform new tasks very quickly by making use of lower‐level skills they have developed when learning previous tasks. Hoekstra, Martens, and Taatgen model this process, showing how a system trained on simple tasks (visual search and two working memory tasks) can then quickly learn to perform the attentional blink task, and it ends up making the same sorts of errors as people do.

## 1 Introduction

Humans have the impressive ability to learn certain relatively simple tasks with minimal instruction and in a very short period of time. The experimental tasks used in (cognitive) psychology are particularly good examples of these types of tasks. Participants have often never encountered these tasks before, yet are quickly able to work out what to do. This quick learning suggests that people reuse previously learned skills and apply them to new contexts (Salvucci, 2013; Taatgen, Huss, Dickison, & Anderson, 2008). For example, if a task requires a stimulus to be remembered for later recall, people do not have to work out how to remember the stimulus, but they can simply reuse the already learned “remembering skill.” It would be unnecessary, in this case, to reinvent the wheel. Learning how to do a new task simply means selecting the appropriate skills, assuming all these skills have already been acquired.

"Skill" is a commonly used term in cognitive psychology and is used to convey many (slightly) different meanings. In the context of this paper, skill refers to the largest unit of procedural knowledge that can be reused in different tasks. There can be many instances in which the same procedural knowledge (i.e., skills) can be reused. Traditionally, the idea of skill reuse was often implemented in cognitive architectures in the form of subgoaling (Newell, 1990). A main task goal could be decomposed in subgoals, each of which could in theory be reused for other tasks. However, the subgoaling mechanism proved to be too brittle to support flexible reuse. Moreover, psychological data turned out to be inconsistent with models using a goal stack (Anderson & Douglass, 2001). The skill‐based approach shares similarities with subgoaling, but it does not use the goal‐stack mechanism.

Reusing skills speeds up learning, but it can also have negative side effects that lead to suboptimal performance even though the cognitive system is, in principle, capable of optimal performance. That is, it is a suboptimal strategy that underlies the impaired performance, not a fundamental information processing limit (e.g., Taatgen, Juvina, Schipper, Borst, & Martens, 2009). One factor underlying the suboptimal strategy choice might be the selection of the wrong skills, either because the "right" skill is not available or because the interpretation of the task cues the wrong skill. A well‐known instance of this is the Stroop effect (Stroop, 1935). Because people are so used to reading words, this automatically triggered skill interferes with the task of naming the color of the word. In this case, words trigger the “reading skill,” which leads to worse performance. Another, less obvious, instance where this can happen is the attentional blink (AB).

The AB is a well‐studied phenomenon in cognitive psychology (Martens & Wyble, 2010). It refers to the finding that the second of two to‐be reported targets in a stream of distractors presented at a rate of 100 ms per item is often missed when it is presented within an interval of 200–500 ms after the first target (T1) (Raymond, Shapiro, & Arnell, 1992). Interestingly, the second target (T2) is hardly ever missed if it is presented directly (i.e., within 100 ms) after the first target (lag‐1 sparing). This suggests that the cognitive system does possess the processing capacity to identify both targets, but that the chosen strategy prevents the second target from being reported.

The crucial aspect of the strategy that most participants use can be the selection of a suboptimal skill to consolidate the targets in memory. Many theories of the AB assume that consolidation of T1 into memory underlies the AB (Akyürek, Abedian‐Amiri, & Ostermeier, 2011). Memory consolidation is thought to be a serial process, meaning that only one consolidation process can occur at a time and that the consolidation has to be completed before a new item can be consolidated. This means that T2 cannot always be consolidated straight away, but sometimes has to wait for the consolidation of T1 to be completed. This leads to the AB when consolidation of T1 has not yet been completed before T2 has disappeared from visual short‐term memory. However, such theories all assume that targets are consolidated as separate memory items, whereas in other areas of memory research, it is assumed that multiple items are consolidated in a single chunk.

The strongest indication that strategy underlies the AB phenomenon is an experiment by Ferlazzo, Lucido, Di Nocera, Fagioli, and Sdoia (2007). In their experiment, participants were instructed to report two target letters (which were always a vowel and a consonant) either separately or as a single syllable. In the latter condition, participants did not exhibit an AB. A possible explanation is that the original instruction cues a strategy in which all targets are consolidated separately, while the syllable instruction encourages consolidation of both targets in a single chunk. We will explore this difference by creating two versions of an AB model that only differ in their consolidation strategy.

To create the model, we have used a novel approach. Instead of creating the model specifically for the AB, we built a model from general skills that we have constructed as parts of other models. In other words, the AB model only links together existing skills. We chose this approach because it mirrors how participants performing an AB task work out what to do. They do not start from scratch, but they tie skills they already possess together in such a way that allows them to perform an AB task.

Many tasks share similarities and, therefore, many tasks require the same skills. The tendency of people to utilize this overlap between tasks calls for the creation of more generalizable models to reflect this approach. Moreover, creating models that use generalizable elements allows for the mechanisms used in these models to be placed in a larger cognitive context because each mechanism is essentially part of a generalizable skill. Currently, phenomena are usually modeled by specific cognitive machinery that captures the phenomenon found in empirical data. It is not common to place this machinery in a larger cognitive context and describe how it relates to other cognitive processes (i.e., specifying “where” or “when” it takes place in cognition). However, this leads to models with very specific mechanisms created to explain findings in one particular experimental paradigm. In contrast, creating models with a skill‐based approach forces the mechanisms to be placed into a larger cognitive context because it is created as a part of a general skill that is also used in other tasks and cognitive processes. Because its underlying mechanism is the same for these tasks, it should also be predictive of behavior outside of the initially modeled paradigm.

We believe that creating cognitive models in this manner can be a promising contribution to the field in general. In particular, because it could aid generalization among the many different models created in the highly specialized and compartmentalized fields of cognitive science. The goal of the skill‐based approach is a continuation of one of the fundamental goals of cognitive architectures. Cognitive architectures have been developed to create a basic framework which can be applied to model a large variety of tasks. This ensures a certain amount of correspondence between models created using the same architecture, independent of the specific task modeled. The skill‐based approach extends this idea. Applying this approach, in combination with a cognitive architecture, will allow for not only the basic architecture to be considered but also previously acquired knowledge (skills).

We created our AB model in the cognitive architecture PRIMs (Taatgen, 2013, 2014). PRIMs (abbreviation for primitive information processing elements) is based on ACT‐R (Anderson et al., 2004) and works in a highly comparable way. The architectures of both ACT‐R and PRIMs consist of a “central workspace” and a number of modules capable of performing specific cognitive functions. The modules can communicate (i.e., exchange the results of their cognitive operations) with each other through the central workspace, which is subdivided in buffers. This exchange of information between the modules in PRIMs is controlled in largely the same way as it is in ACT‐R. In ACT‐R, this is done by productions, and in PRIMs it is done by operators, but they have similar functionalities. For a more extensive discussion of PRIMs, see Taatgen (2013). A crucial difference between ACT‐R and PRIMs is that in PRIMs operators are by default further organized into skills. A skill is a collection of operators capable of accomplishing a certain goal or processing step. Skills, therefore, form the bridge between single operators and the complete task that is being modeled. A task is built up from a certain number of skills and a skill, in turn, consists of a certain number of operators. This distinction is helpful because it allows for more flexibility while maintaining a high level of organization. Flexibility is improved because the model can diverge from the beaten path if the situation asks for it. Additionally, it allows for connections between operations that cannot be executed in a single operator (e.g., because they use the same buffers).

Skills are combined into tasks by instantiating variables that are part of the skill (and, in turn, the underlying operators). For example, a skill for visual search may be instantiated by the type of item that we search for. Variable instantiation is also used to link skills together. For example, the visual search may need a more elaborate criterion, which in itself is another skill (e.g., search for a sheep with five legs). Therefore, building a task model that can be composed of existing skills entails instantiating the variables in those skills (and nothing else).

The generalizability of skills makes it possible to use the same skills in models of different experimental tasks. The organization into skills thus allows us to employ a novel approach to constructing cognitive models, placing them in a context of related models, tasks, and skills. We had two main goals in this project. First, we wanted to investigate the feasibility of creating a cognitive model by tying together general skills. Second, we wanted to create a model of the AB which is capable of capturing the most commonly found effects in the AB paradigm, including differences due to instruction.

## 2 Methods

Instead of creating operators specifically for the AB, we first considered which general skills are required to perform an AB task and assembled the AB model from these skills. In other words, we assembled the model from skills (we assume) participants have already acquired before entering the laboratory.

Based on previous work and other models of the AB, we identified four basic skills (cognitive processing steps) which had to be performed by our model of the AB. In short, these four skills are visual search, consolidation, retrieve, and report. More extensive discussions of them will follow. We developed these four skills by first creating models of other tasks which share (some) of these same basic skills. This step was done to get a better idea of what these general skills should be capable of and to test the plausibility of these skills.

First, we will describe the three models that provided the building blocks for the AB model. These three models are as follows: (a) a visual search model, (b) a model of a simple working memory (SWM) task, and (c) a model of a complex working memory (CWM) task. Not all parts of all three models will be used for the AB model, but all three contain at least one of the four basic skills needed to perform an AB task.

**Fig. 1:**
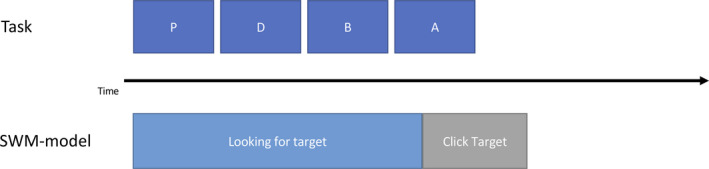
Visual representation of the visual search model. The model uses the “Look for target” skill to discriminate between non‐targets (consonants) and the target (vowels) and clicks on the target when it discovers one. The search skill determines the category by a memory retrieval and is reused in both attentional blink models.

The first model, the visual search model, is very straightforward. The goal of this model is to find a vowel on a screen filled with other letters (see Fig. 1). The main search skill processes the current visual item and determines its category through memory retrieval. If it does not match the target category (vowel in this case), it transfers control to another skill which focuses on the next search item. In visual search, this is a shift of attention to another item. If it does match the target category, it transfers control to a third skill, in this case a skill that clicks on the target with the mouse. Finally, if it runs out of items to attend to, it transfers control to yet another skill, which is not instantiated in the visual search model.

**Fig. 2:**
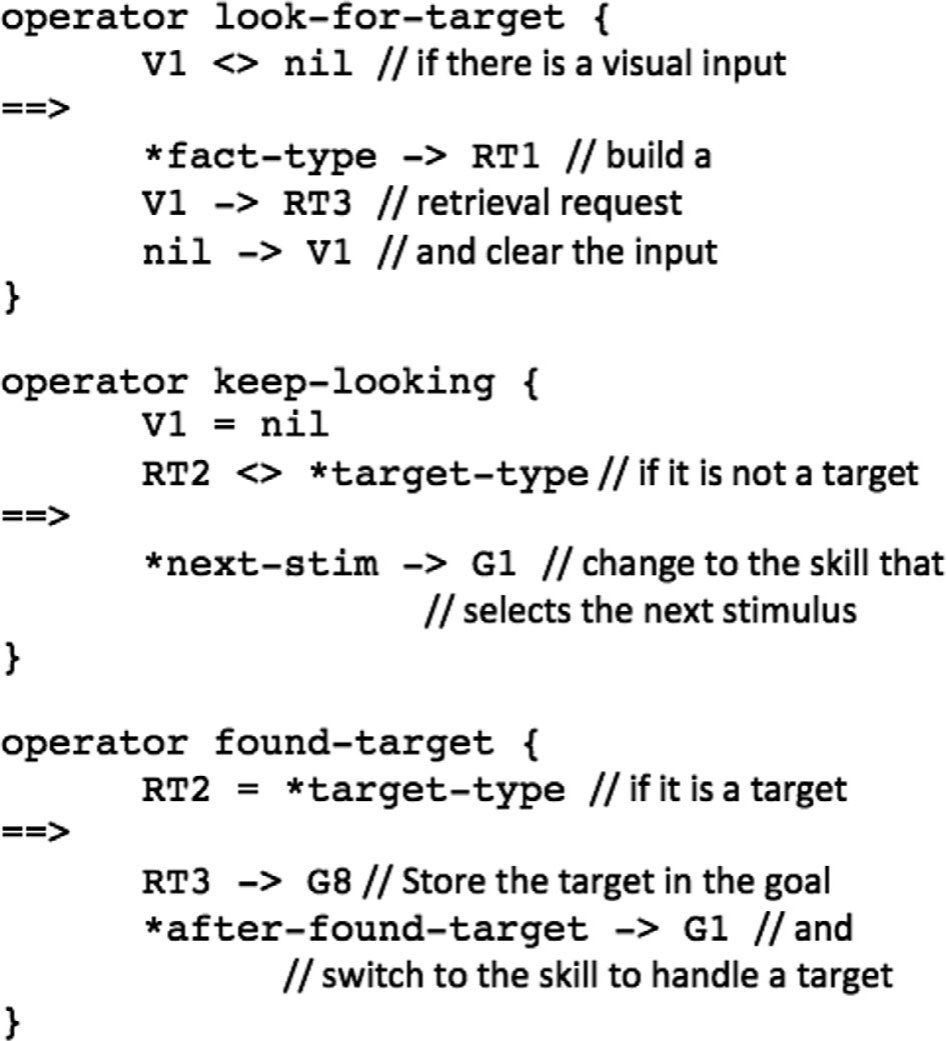
Example operators of the visual search skill.

In the AB model, we will reuse the search skill to find targets, but we will instantiate it differently. To illustrate, Fig. 2 lists the operators that make up the search skill, slightly abbreviated for clarity. In these operators, V *x* refers to a slot in the visual buffer, RT *x* refers to a slot in the retrieval (declarative memory) buffer, and G *x* refers to a slot in the goal buffer. In these operators, values that are preceded by an asterisk are variables that need to be instantiated for a particular task. For visual search in the context of the previously mentioned example, we instantiate *fact‐type with vowel, *next‐stim with the attend‐next skill, and *after‐found‐target with the click‐item skill.

The second and third basic models are strongly related and provide the final basic skills. Both models deal with working memory tasks which require the participants to remember presented items and, after presentation of the items, recall which items have been seen. Although they both include a consolidation step, they accomplish this step with a different skill. Both build a chunk in working memory; however, they differ in the moment of consolidation. The “consolidate‐separate” skill, used in the CWM model, starts consolidation immediately after an item is encountered. In contrast, the “consolidate‐chunk” skill, used in the SWM model, only starts consolidation after all items have been presented. Using these two consolidation skills, we created two versions of the AB model, a “consolidate‐separate” version and a “consolidate‐chunk” version.

**Fig. 3:**
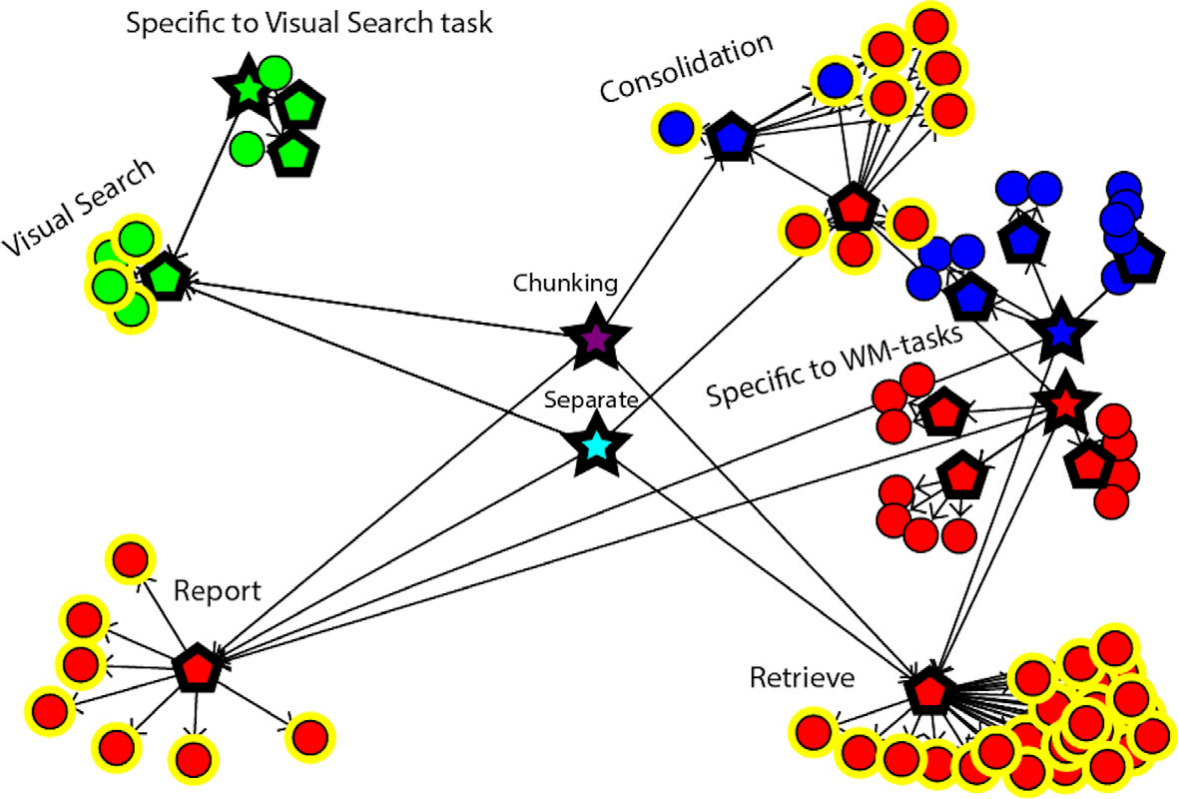
Visual depiction of the two versions of the attentional blink (AB) models and the basic models from which they are assembled. The “consolidate‐chunk” AB model is represented by the purple star, and the “consolidate‐separate” AB model is represented by the light blue star. Both of the AB models are only built up from skills originating from the three basic models. Furthermore, the “consolidate‐chunk” version of the AB model takes its consolidation skill from the simple working memory task (in blue) and the “consolidate‐separate” version of the AB model takes its consolidation skill from the complex working memory task (in red). A yellow circle around a skill indicates that this skill is used in both a basic model and one of the AB models.

Finally, these two working memory task models provide the “retrieve” skill and the “respond” skill. The “retrieve” skill retrieves the appropriate consolidated item from memory and the “response” skill gives the appropriate response based on the retrieved item, completing the skills needed to create the AB model (Fig. 3).

**Fig. 4:**
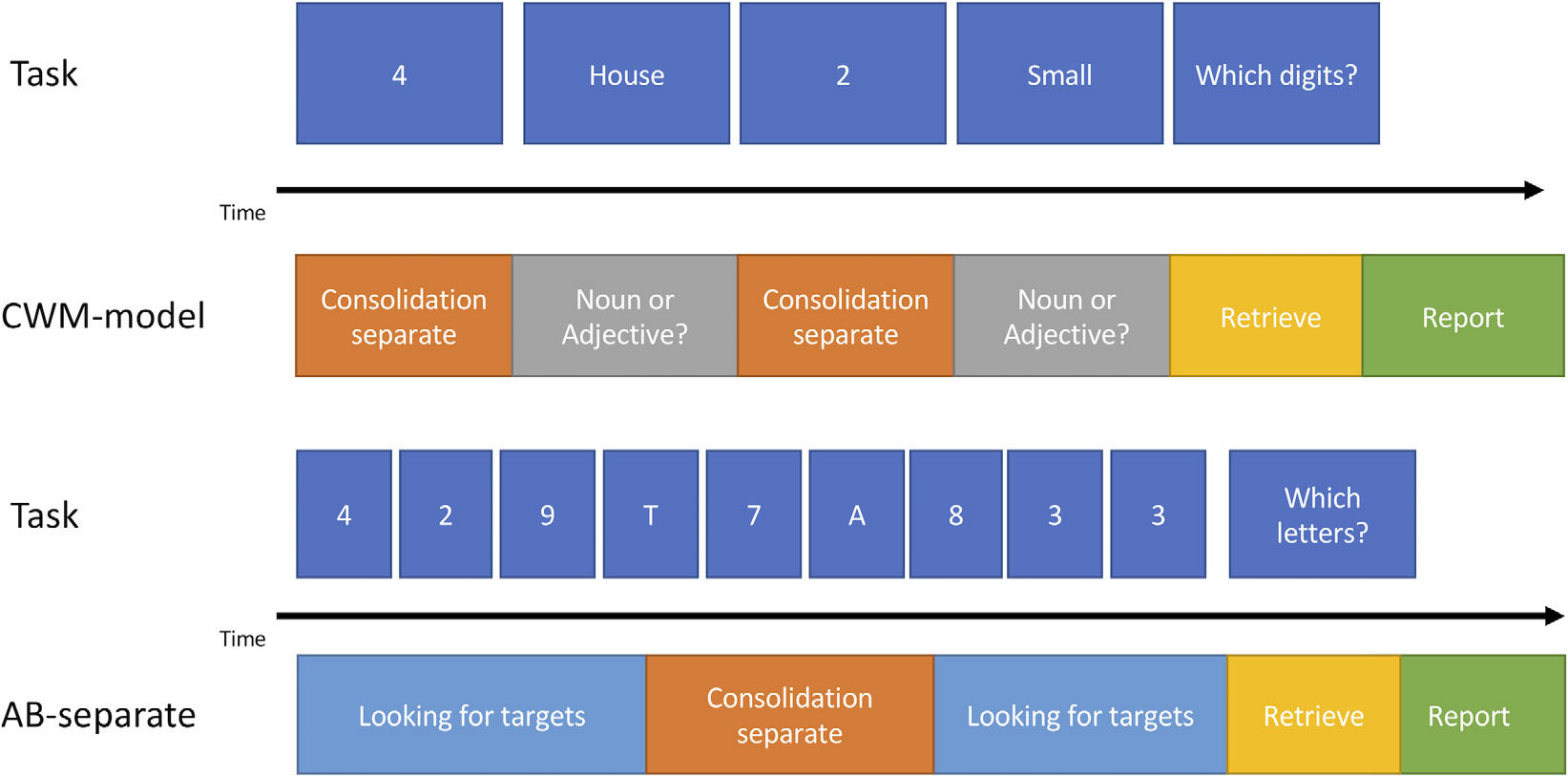
Visual representation of the complex working memory (CWM) model and the version of the attentional blink (AB) model that uses the same consolidation skill, the “consolidate‐separate” version. The CWM model consolidates the presented numbers separately because working memory is engaged by the secondary task. The “consolidate‐separate” AB model starts consolidation directly after detecting T1 and therefore misses T2 on the lag 2 trial pictured here.

**Fig. 5:**
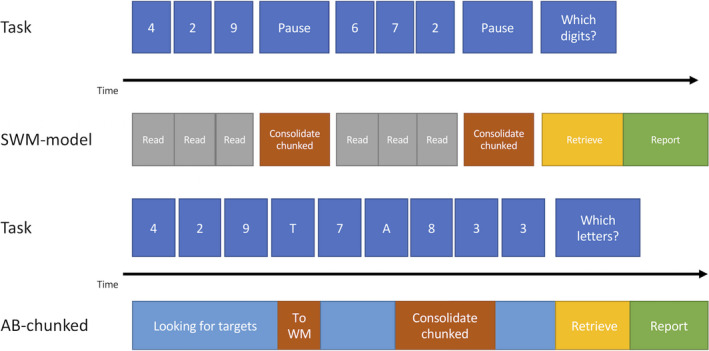
Visual representation of the simple working memory (SWM) model and the version of the attentional blink (AB) model that uses the same consolidation skill, the “consolidate‐chunked” version. The SWM model consolidates the three numbers presented before a pause as one chunk. The “consolidate‐chunked” version of the AB model moves T1 into working memory directly after detecting it and only consolidates both targets after detecting T2. This leads to the successful consolidation of both targets even at the Lag 2 trial pictured here.

The four skills described above form the basic building blocks of both versions of our AB model. To finalize the AB model, the basic skills were put together in one model and were instantiated to fit the context of an AB trial. This procedure was the same for both versions of the AB model. In the AB model, after presentation of a stimulus, the “search” skill checks, whether this is a target or a distractor. In other words, the *fact‐type variable is instantiated with letter. If the stimulus is a distractor, it is ignored and the model waits for the next stimulus (*next‐stim is instantiated with wait). If the stimulus is a target, it switches to the consolidate skill (by instantiating *after‐found‐target with that skill) that moves the stimulus into a working memory slot. The consolidate skill is the source of the AB in our model. Depending on which skill is used to accomplish consolidation, the model either starts consolidating directly after encountering the first target or postpones consolidation until the second target is encountered. If the chunk is consolidated, no other operator can be executed for a period of, on average, 200 ms (the imaginal delay parameter in ACT‐R), leading to a possible AB (see Fig. 4). If consolidation is postponed until the arrival of the second target, no AB will occur at this point and the model will keep performing the task normally (see Fig. 5). After all stimuli are presented, the model will retrieve the targets that were consolidated on this trial (the “retrieve” skill) and then, after the retrieval, responding to the retrieved items (the “respond” skill).

## 3 Results

We compared the behavior of the models with human performance. This was done to verify the feasibility of the basic models and to check how well the final AB model could model the AB phenomenon. The comparisons were made with existing data from the literature, except for the visual search model as we had found no suitable data to compare it with. This is likely due to the fact that our visual search model is very simple and does not have any other functionalities besides what is described in the method section. Furthermore, the visual search model was not our primary interest, as it is not responsible for creating the AB.

For the SWM model, a specific task was modeled, requiring participants to remember a certain number of digits and report them at the end of a stream (Anderson, Bothell, Lebiere, & Matessa, 1998). The critical manipulation in this experiment was that the digits were presented in multiple groups. This grouping was thought to influence chunking of the digits, digits grouped together during presentation would also be grouped together in memory (i.e., chunked together). The findings supported this expectation such that participants showed longer reaction times during recall for the first item of a group, indicating that the groups were remembered (and recalled) as one chunk. The data from the SWM model showed this same pattern in reaction times as reported in Anderson et al. (1998). Just as in the reported data, reaction times in our model to items relating to the start of a new chunk were significantly longer, as tested by linear regression (β = 0.43, *SE* = 0.003, *t* = 128.7, *p* < .001).

**Fig. 6:**
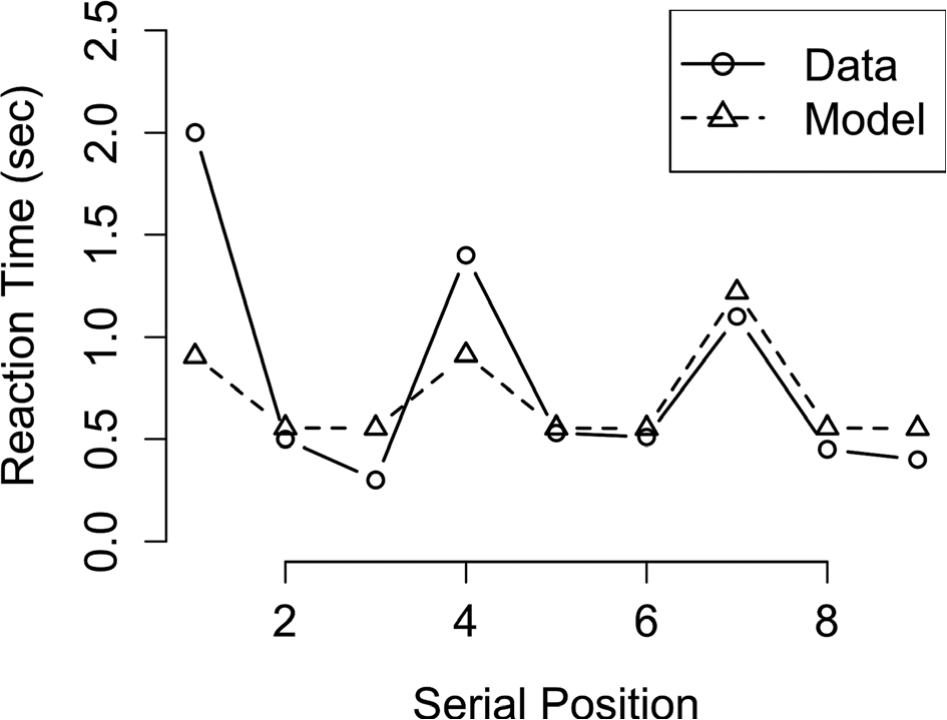
Model fit for reaction times in the simple working memory task. Figure depicts the retrieval thresholds produced by the model (dashed line) and human data (solid line).

As can be seen in Fig. 6, the reaction times produced by the model show the same typical pattern as the human participants. This reflects the strategy used by the model (and presumably the participants) of recalling the remembered digits. The digits are stored in memory in chunks of three, and this influences how the recall occurs. First, the full chunk containing all three digits is retrieved from memory and, subsequently, the three responses are given without any further memory retrieval. Note, however, that the model is unable to capture the extra‐long reaction times at the start of the recall‐phase. These increased reaction times are likely due to processes relating to getting started on a new task, an aspect of the task unrelated to working memory so we chose not to model it at this moment. In addition to the reaction times, we also compared the accuracy of our model with the accuracy as reported in the original study (not pictured). The original paper only reports a significant effect of the length of the to‐be‐learned list (*F*(9,621) = 128.05; *p* = .001) in the direction that longer lists are harder to recall. Their data also show a clear effect of serial position, in that earlier presented letters are recalled more accurately than later presented letters (although there is also a small recency effect). We tested our model on these same two effects of list length and serial position with a linear mixed effect model. Our AB model shows a significant effect of both list length (β = 0.05, *SE* = 0.01, *z* = 3.7, *p* < .001) and serial position (β = 0.13, *SE* = 0.04, *z* = 3.4, *p* < .001), which is in line with the data presented in the original study.

The SWM model data were collected over 15 runs with 350 trials per run (total of 5,250 trials). Most of the parameters were the default PRIMs parameters (which are the same as the ACT‐R default parameters for a big part). Only the retrieval threshold (rt was 0.6 instead of 0) and the latency factor (lf was 0.15 instead of 1.0) differed from default. This latency factor of 0.15 was also used in a previous model of the AB (Taatgen et al., 2009).

Second, we will discuss the comparison between the CWM model and human performance. In the task we modeled, a series of 3, 4, 5, or 6 digits were presented to the model. In between presentation of the digits, the model did a word‐decision task in which it had to distinguish between nouns and adjectives. We compared the performance of our model on this task to a similar experimental task (Daily, Lovett, & Reder, 2001). In this task, participants were instructed to remember a series of digits (also 3–6), but here the digits were presented among letters which they were required to read aloud. Both of these tasks have in common that working memory is required to perform the interrupting task (either deciding between a noun or adjective or reading a letter aloud). This demand on working memory makes it impossible for the participants (and the model) to chunk the items in memory.

**Fig. 7:**
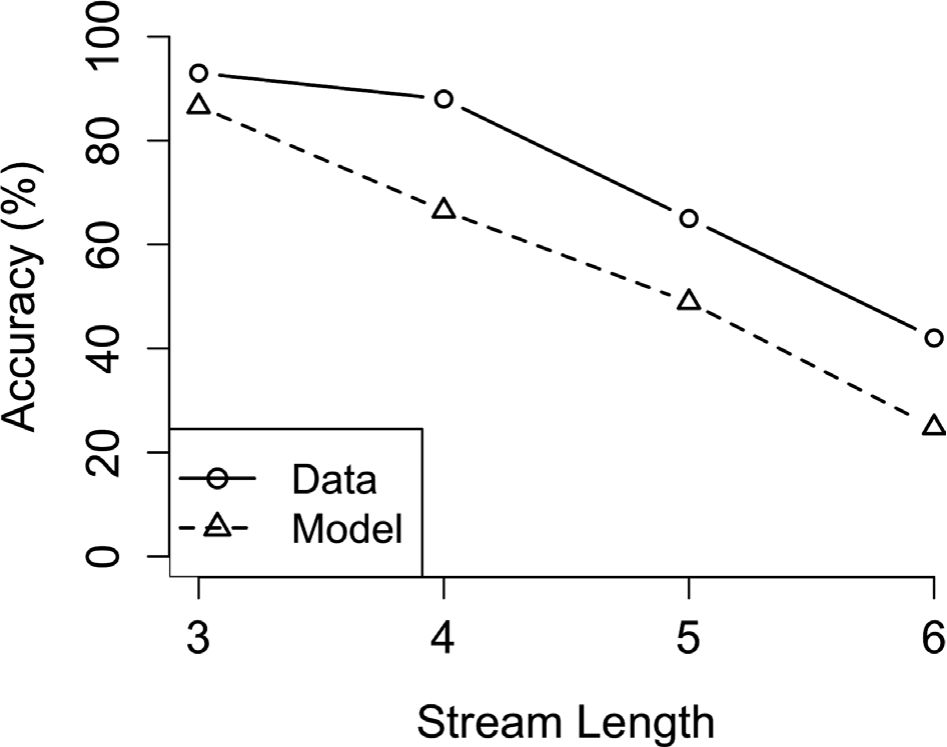
Complex working memory model fit for accuracy data. Depicted is the average accuracy as a function of list length for the model (dashed line) and the human data (solid line).

We compared model performance with human performance with respect to accuracy (see Fig. 7). This was the only measure we could use because the original paper did not report any other measure (e.g., reaction times). Generally, the model shows a good fit to the human data reported by Daily et al. (2001). Both the model and the participants show decreased accuracy when the length of the presented list is longer. In both the original data (*F*(3, 63) = 90.80, *p* = .0001) (tested with an anova) as in our model (β = −0.2, *SE* = 0.005, *t* = −37.2, *p* < .001) (tested with a general linear model), this was a strongly significant result. This decreased accuracy for longer lists occurs in the model because the presentation of the longer lists takes a longer time to be completed. The longer time required for presentation allows for additional item‐decay in memory, leading to reduced accuracy for longer lists. The model, however, generally underestimates accuracy, this is probably due to the model being unable to capture the primacy effect (Murdock, 1962). The primacy effect is often modeled by including a rehearsal mechanism. The fact that we did not include such a mechanism to the model could thus explain the general underestimation of the accuracy. Rehearsal is not directly related to working memory consolidation, so for reasons of simplicity we did not include this process in the model. The SWM model data consist of a total of 4,500 trials (15 runs with 300 trials per run). Similar to the CWM model, most of the parameters were kept at the default setting, except for the retrieval threshold and the latency factor. The retrieval threshold had a value of 0.5 and the latency factor was set at 0.15 for our model.

**Fig. 8:**
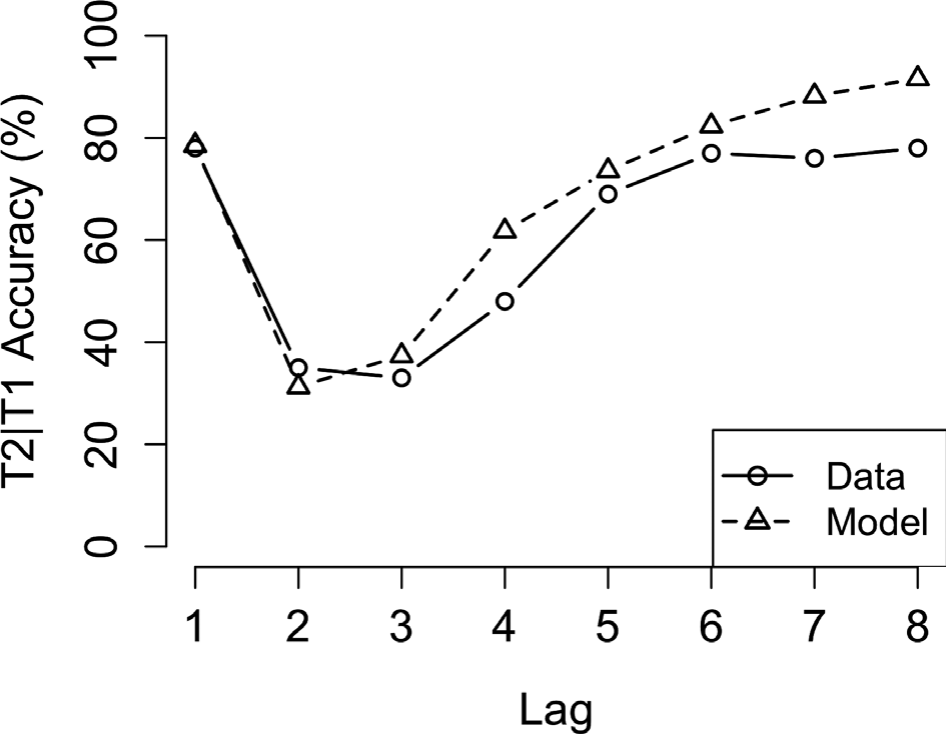
Attentional blink (AB) model fit for T2 accuracy. Figure showing T2 accuracy in an AB task for the model (dashed line) and human data (solid line).

Finally, we compared our AB model (which resulted from the combination of the above discussed models) with human AB performance (see Fig. 8). The specific task we modeled was the classic AB task reported in Chun and Potter (1995). In this standard version of the AB, participants are instructed to identify two digits within a stream of distracting letters and, at the end of the stream, report which digits they have seen. We modeled this experiment with the version of the AB model that used the “separate‐consolidation” skill. The AB, characterized by a strong performance decrement at lags 2 and 3, is nicely captured by our AB model. Performance at Lag 2 (β = −0.47, *SE* = 0.02, *t* = −25.2, *p* < .001) and Lag 3 (β = −0.41, *SE* = 0.02, *t* = −22, *p* < .001) is significantly lower than at Lag 1. The original paper also reports significant performance decreases at Lag 2 (*t*(5) = 6.6, *p* < .01) and Lag 3 (*t*(5) = 4.1, *p* < .01). In the model, the AB occurs because consolidation of the first target (T1) is still in progress when the second target (T2) is presented. Therefore, T2 cannot be consolidated and will not be reported at the end of the stream. Our model also shows the typical lag‐1 sparing effect. This is because consolidation of T1 often has not started at the moment that T2 is presented at lag 1. Therefore, they can both be consolidated into a single chunk and reported at the end of the stream. Finally, the model shows the slow performance increase for the later lags (lag 4 and higher). This is caused by the slow increase of the likelihood that T1 consolidation is finished by the time T2 is presented.

The AB model data consist of 15 runs with 600 trials per run (for a total of 9,000 trials). Most of the parameters were kept at the default values, except for the latency factor, activation noise, and the imaginal delay factor. The latency factor was again set to 0.15, following Taatgen et al. (2009). The activation noise was set to 0.35, which is a little bit higher than default; this was done to create some extra noise on the behavior of the model and produce slightly more human‐like performance. Finally, the time it took for a chunk to be encoded into the imaginal buffer, the imaginal delay parameter, varied from trial to trial (also following Taatgen et al., 2009). Its value ranged from 50 to 350 ms and was drawn from a uniform distribution with an average of 200 ms. This variation made it possible for the model to still detect both targets on lag‐2 and lag‐3 trials in some instances.

**Fig. 9:**
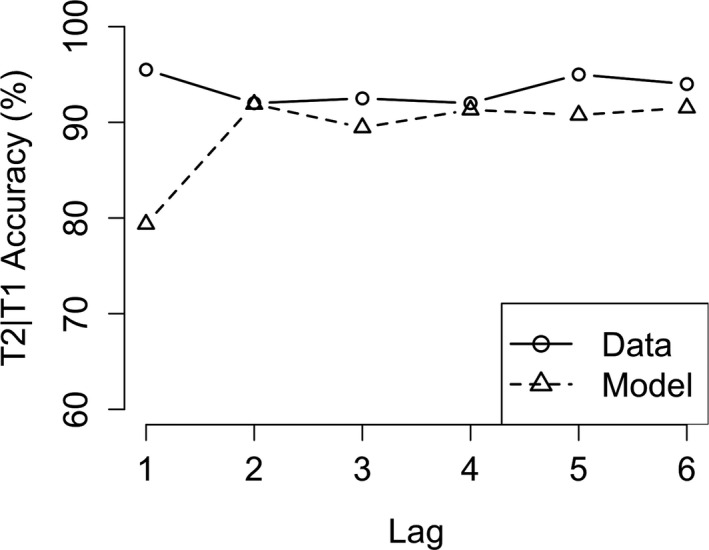
Model fit for the alternative attentional blink (AB) model (consolidate‐chunk). Figure showing T2 accuracy for the alternative AB model (dashed line) and human data (solid line).

Using the other version of the consolidation skill (the “consolidate‐chunk” version) in the AB model, however, will prompt the model to always try to consolidate both targets into a single chunk, which should prevent the AB to occur. Importantly, both versions of the AB model were run with exactly the same model parameters. We compared the performance of the AB model instantiated this way to the data from Experiment 2 in the paper reporting a reduced AB when participants were instructed in a way that promoted chunking (Ferlazzo et al., 2007; see Fig. 9). The model mirrored the general performance level and, crucially, showed no AB. Performance on lag 2 (β = 1.76, *SE* = 0.15, *z* = 11.6, *p* < .001) and lag 3 (β = 1. 6, *SE* = 0.14, *z* = 11.6, *p* < .001) was significantly higher in the “consolidate‐chunk” version compared to the “consolidate‐separate” version. The model, however, shows a slight performance decrease at lag 1 which is significantly lower than lag 6 performance (β = −0.13, *SE* = 0.01, *t* = −2.1, *p* < .001). This was caused by the way in which noise in the visual system was simulated, which meant that occasionally T2 had already disappeared before it was processed fully and therefore it was missed. We do not consider this problematic, because in many AB experiments lag 1 performance is slightly lower than performance on long lags. The “consolidate‐chunk” version model data also consist of 15 runs with 600 trials per run (in total 9,000 trials). As was mentioned before, the parameters were exactly equal to the parameters used for the “consolidate‐separate” version of the model.

## 4 Discussion

Computational models of cognitive psychological phenomena are often able to accurately capture one specific phenomenon; however, they are often hard to generalize to other tasks and cognition in general (Anderson et al., 2004). In this paper, we attempted to (partly) bridge this gap by employing a novel approach to building cognitive models, which mirrors the way people approach a new task. People do not consider every task in isolation, but they use knowledge gained from the past. That is, they reuse skills learned from doing other tasks and apply them to the (new) task at hand (Salvucci, 2013; Taatgen, 2014). This paper describes our attempt to apply a similar approach. However, our approach differs from earlier efforts because the “building blocks” for a task are much larger. In the earlier work, the level of reuse was operators. Therefore, a new task had to be constructed out of existing operators, which is, even in the case of a simple task such as the AB, still a substantial number (see Fig. 3). In the skill‐based approach, only four skills needed to be combined. This is not only consistent with the amount of time people need to prepare for the task, but it also sheds new light on the nature of the AB and the effect of instruction.

The comparisons between our models and human data show that our models are reasonably able to capture human performance. This result demonstrates the basic feasibility of the described modeling approach. It is possible to break a task down into a limited set of skills that are reusable in different tasks. This is an important first step toward creating more generalizable models because it allows for a method of creating models that are built from the same building blocks. Using existing building blocks when modeling a new task allows for much more integration of any new model into the already existing collection of models and might better reflect the way people approach a new task. Finally, our AB model can be placed in a larger cognitive context. It provides a clear and generalizable explanation of the AB using terms that can be related to other theories and empirical findings. This is the main benefit of creating models with the skill‐based approach. In our case, our model not only provides an account of the AB but, importantly, also suggests some general limitations to memory consolidation. The skill‐based approach facilitates the investigation of the mechanisms that lead to experimental findings instead of only focusing on the findings themselves.

Note, however, that the devil is in the details. Building a model using this approach can be challenging, especially when it comes to determining how small differences between tasks can best be handled. Such differences make it difficult to use exactly the same operator (and therefore the same skill). Every operator has a condition‐checking part (which checks whether this operator should be activated now) and an action‐performance part (which actually executes the “cognitive action” or PRIM). The action‐performance part is relatively easy to generalize across tasks, but the condition‐checking part is more challenging. Basically, the condition‐checking part checks whether the situation matches the predefined situation in which this operator should be executed. This makes it difficult to generalize the condition‐checking across tasks since a different task usually also means a different situation. We solved this problem in the models described in this paper by defining the conditions in such a way that they work for all the modeled tasks. This is a workable solution, but it is time‐consuming and a more optimal method for condition‐checking is needed.

A further limitation to the models described here is that they did not perfectly capture all aspects of human performance. However, we do not see this as a major issue because we did not set out to create complete models of the described experimental paradigms. Instead, we aimed to create models of the main findings only because we were merely interested in the skills that are important for the AB. Although there remain limitations and improvements to be made to the skill‐based approach, we consider it a feasible and promising approach to improve the generalizability of models.

The second goal we set out to achieve in this paper was to create a model of the AB that can account for differences due to instruction. The model described in this paper produces most of the basic effects from the classic AB task, showing lag‐1 sparing, the AB itself, and the gradual improvement on later lags. Although there are many additional aspects of the AB reported in the extensive literature which we did not discuss, we believe that the model described here is an adequate first attempt that can be built on in future work.

For now, the fact that the model captured the basic AB effects implies that these effects, at their core, may be caused by improper selection of skills. At the start of a new task, a participant has to figure out which skills to combine to be able to perform the new task. The models we created suggest that there are (at least) two different skills which can take care of the consolidation into working memory aspect of the task: (a) consolidate every presented target into working memory separately (as in the CWM task) or (b) consolidate targets as larger chunks (as in the SWM task). The chunk‐consolidation skill as used in the SWM task would be the optimal pick in this situation; two items can be consolidated into one chunk and there would be no negative unexpected effects. This approach is perhaps employed by participants after receiving the experimental instructions from the Ferlazzo et al. (2007) study. However, given that standard AB instructions consider targets as separate items probably prompts most participants to use the separate‐consolidate skill from the CWM task.

The emphasis put on strategy by our model could explain previous findings in the AB literature that have proven difficult to explain. This includes the effect of instructions as well as the existence of non‐blinkers (individuals who do not show an AB; Martens, Munneke, Smid, & Johnson, 2006; Willems & Martens, 2016; Willems, Wierda, van Viegen, & Martens, 2013), and the reduction of AB magnitude because of training (Choi, Chang, Shibata, Sasaki, & Watanabe, 2012). All these effects could be explained by the type of consolidation strategy. Different instructions might cue the “correct” consolidation skill, non‐blinkers could be more naturally inclined to use the “correct” chunking strategy compared to blinkers, and the training procedure by Choi and colleagues might nudge participants toward using the same optimal strategy.

To summarize, our novel skill‐based approach to cognitive modeling resulted in valid models, created using a more natural and human‐like method. In addition, we believe it shows great potential to generate more generalizable and thus more flexible models. Therefore, we will continue working on the skill‐based approach, by testing the underlying assumptions (e.g., people are able to apply previously learned skills to new tasks) and creating additional models using this approach for other paradigms. Finally, building models with the skill‐based approach can lead to interesting new perspectives on well‐established cognitive phenomena such as the AB. The choice of consolidation strategy may play an important role in the AB, explaining individual differences as well as instruction and training effects of the AB.
